# Case Report: Anomalous origin of the left main coronary artery arising from the left ventricular outflow tract

**DOI:** 10.3389/fcvm.2025.1640534

**Published:** 2025-08-08

**Authors:** Huynh Thi Minh Thuy, Tran Minh Bao Luan, Pham Tho Tuan Anh, Nguyen Hoang Dinh

**Affiliations:** ^1^ Diagnostic Imaging Center, Tam Anh General Hospital, Ho Chi Minh City, Vietnam; ^2^ Department of Cardiovascular and Thoracic Surgery, School of Medicine, University of Medicine and Pharmacy at Ho Chi Minh City, Ho Chi Minh City, Vietnam; ^3^ Thoracic and Vascular Surgery Department, University Medical Center Ho Chi Minh City, Ho Chi Minh City, Vietnam

**Keywords:** congenital coronary artery, left main coronary artery, anomalous origin of the coronary artery, coronary computed tomography angiography, coronary angiography

## Abstract

An anomalous origin of the left main coronary artery arising from the left ventricular outflow tract is an exceedingly rare congenital coronary anomaly, typically associated with impaired myocardial perfusion. Here, we report the case of a 67-year-old asymptomatic woman in whom an anomalous origin of the left main coronary artery, arising from the left ventricular outflow tract below the aortic valve, was incidentally identified during routine preoperative cardiac evaluation. Despite the anatomical abnormality, the patient exhibited no clinical or imaging evidence of myocardial ischemia. This finding is likely explained by a marked dilation of the right coronary artery and the presence of well-developed collateral circulation supplying the left coronary system. With no evidence of ischemia and maintained ventricular function, a non-interventional approach was deemed appropriate. This case highlights the importance of comprehensive anatomical and functional assessment in detecting clinically silent coronary anomalies and underscores the value of advanced cardiac imaging in the preoperative evaluation of elderly patients undergoing non-cardiac procedures.

## 1 Introduction

Congenital coronary artery anomalies are rare but clinically significant entities due to their association with myocardial ischemia, arrhythmias, and sudden cardiac death. Among these, an anomalous origin of the left main coronary artery (LMCA) arising from the left ventricular outflow tract (LVOT) is exceptionally rare, with only a few cases reported and not yet systematized in the literature. In normal anatomy, the LMCA arises from the left coronary sinus of Valsalva, above the aortic valve. An origin from the LVOT introduces unique hemodynamic implications due to its subvalvular location. The size of the LMCA ostium, the degree of right coronary artery (RCA) dilation, and the presence of well-developed collateral circulation supplying the left coronary system all play important roles in determining myocardial perfusion. This report describes an incidental finding of a hypoplastic and anomalous LMCA originating from the LVOT in a 67-year-old asymptomatic woman.

## 2 Case presentation

A 67-year-old woman was admitted for elective lumbar spine surgery to treat degenerative disc disease. Her family medical history did not reveal any congenital disorders or cardiovascular disease. She had no history of cardiovascular symptoms and no prior diagnoses of coronary artery disease, hypertension, or diabetes mellitus. Physical examination findings were unremarkable for cardiac disease, with normal vital signs (heart rate: 72 bpm; blood pressure: 121/72 mmHg), and no dysmorphic features or respiratory distress. Cardiac auscultation revealed regular heart sounds without murmurs. Routine preoperative transthoracic echocardiography demonstrated normal intracardiac structures and preserved biventricular function, with left ventricular ejection fraction (LVEF) 65%, left ventricular end-diastolic volume (LVEDV) 80 mL, left ventricular end-systolic volume (LVESV) 28 mL, fractional area change of the right ventricle (FAC_RV) 41%, tissue doppler imaging systolic velocity (TDI S') 13.7 cm/s, tricuspid annular plane systolic excursion (TAPSE) 19 mm, and systolic pulmonary artery pressure (sPAP) 17 mmHg. However, it revealed a dilated RCA, and the LMCA was not visualized. An unusual flow signal near the aortic root raised suspicion for an anomalous coronary origin. On both short- and long-axis views of the aortic valve, color Doppler imaging revealed a jet at the commissure between the left coronary cusp and the non-coronary cusp (Supplementary Video S1). A baseline electrocardiogram showed no significant abnormalities. Relevant laboratory results were within normal ranges (high-sensitivity troponin T: 13 ng/L, NT-proBNP: 69 pg/mL, white blood cell count: 11.3 × 10^9^/L, red blood cell count: 4.6 × 10^12^/L, hemoglobin: 13.5 g/dL, C-reactive protein: 4.1 mg/L, and estimated glomerular filtration rate: 87.2 mL/min/1.73 m^2^).

**Figure 1:**
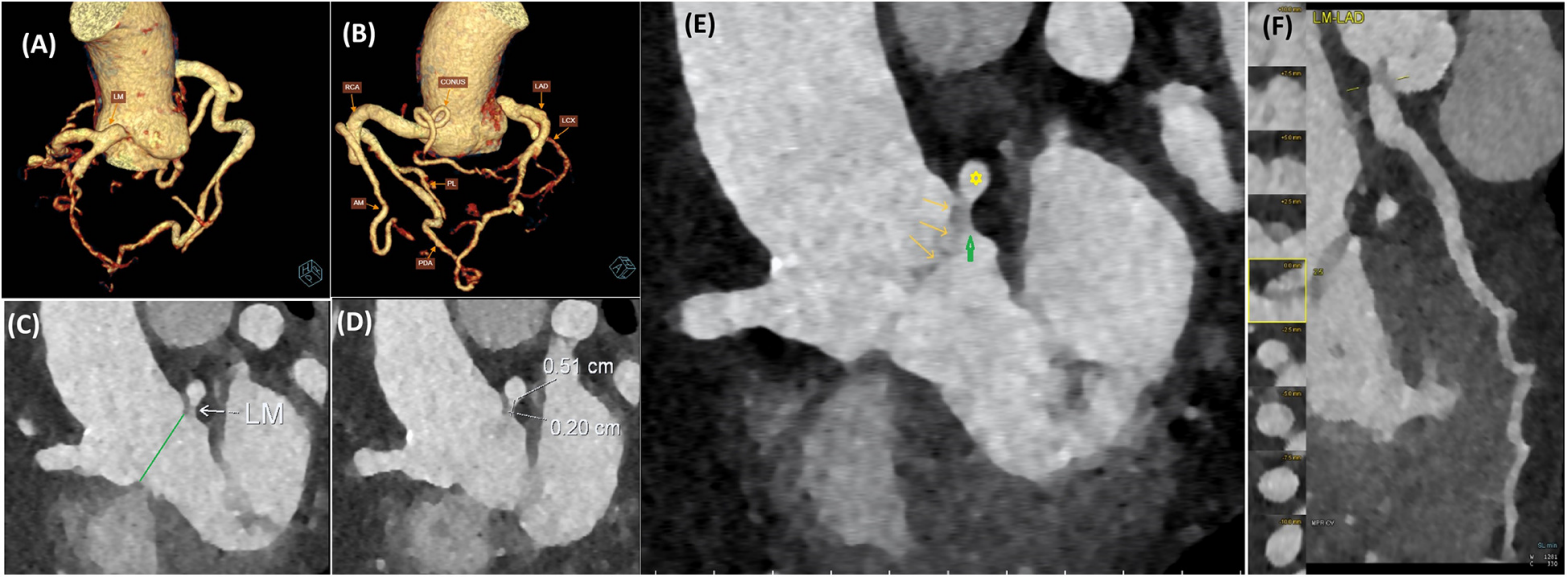
Coronary computed tomography angiography shows the origin of the LMCA below the aortic valve (originating from the LVOT). **(A)** 3D CT reconstruction shows the LMCA origin below the aortic annular plane at the commissure between the left coronary cusp and the non-coronary cusp. **(B)** 3D CT reconstruction shows a dilated RCA and collateral circulation from the RCA to the left coronary system. **(C)** The LMCA originated below the aortic annular plane (green line). **(D)** Ostial narrowing and the intramural segment of LMCA measurements. **(E)** Relationship of LMCA trunk (yellow asterisk) and hypoplastic ostium (green arrow) to aortic valve annulus and aortic valve leaflets (yellow arrows). **(F)** LMCA-LAD artery course reconstruction. LM, left main coronary artery; LAD, left anterior descending artery; LCX, left circumflex artery; RCA, right coronary artery; CONUS, conus branch of the right coronary artery; AM, the acute marginal artery; PDA, posterior descending artery; PL, posterolateral branch artery.

Coronary computed tomography angiography (CCTA) confirmed that the LMCA originated below the aortic annular plane at the commissure between the left coronary cusp and the non-coronary cusp, with a 5 mm transmural course and a 2 mm ostial narrowing (Figure 1), consistent with hypoplastic features. The downstream LMCA segment and RCA were dilated. The LMCA gave rise to the left anterior descending (LAD) and left circumflex (LCx) arteries, both of normal caliber (Figure 1).

**Figure 2:**
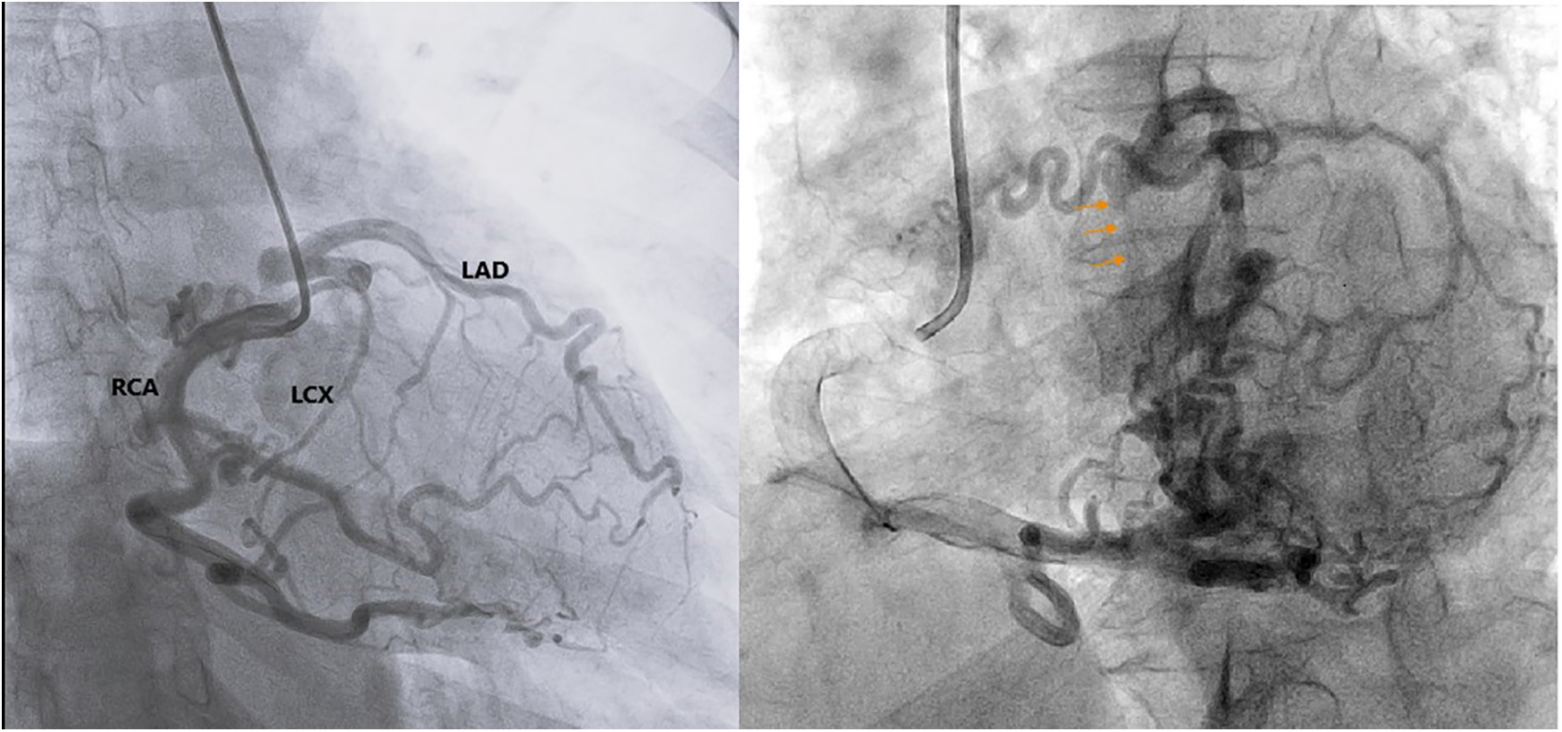
Coronary angiography confirms the LMCA originating below the aortic annular plane, evidenced by retrograde contrast flow from the LMCA into the LVOT (orange arrows). LAD, left anterior descending artery; LCX, left circumflex artery; RCA, right coronary artery.

The patient subsequently underwent coronary angiography to confirm the diagnosis. Angiographic images revealed multiple tortuous collateral vessels originating from a dilated RCA, draining into the LAD, and subsequently into the LCx. The conus branch of the RCA, the acute marginal, posterior descending artery, and posterolateral branches were observed to contribute collateral flow into the LAD (Figures 1, 2). The LMCA was confirmed to originate below the aortic annular plane, evidenced by retrograde contrast flow from the LMCA into the LVOT (Figure 2, Supplementary Video S2). There were no other lesions identified in the LAD, LCx, or RCA arteries.

The patient was asymptomatic and had preserved left ventricular systolic function with no regional wall motion abnormalities and no signs of ischemia on stress echocardiography. Given the absence of symptoms and the preserved cardiac function, and in light of the patient's refusal to undergo cardiac surgery, no further cardiovascular intervention was pursued. The patient subsequently underwent a successful spine surgery without perioperative cardiac complications. During a 1-year follow-up, the patient underwent routine clinical evaluations, serial electrocardiograms to monitor for ischemia, and echocardiography at 6-month intervals to assess cardiac function. No cardiovascular complications were observed, and the patient remained in a stable condition with preserved overall health.

**Figure 3:**
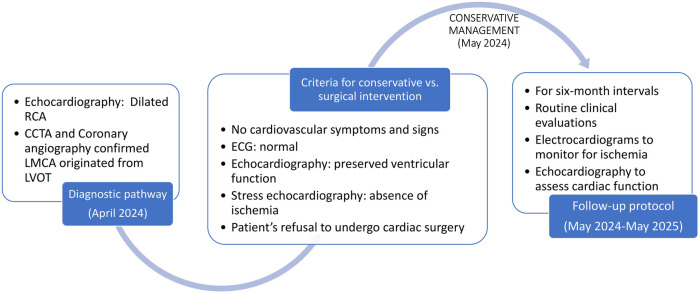
The timeline of the case.

The timeline of the diagnosis and treatment of the patient is shown in Figure 3.

## 3 Discussion

Congenital anomalies of the coronary arteries are rare, with a reported incidence of approximately 0.2%–1.4% in the general population undergoing coronary angiography. Anomalies involving origin, course, and termination of the coronary arteries have been described extensively. However, the specific anomaly of the LMCA arising from the LVOT is exceptionally rare, with only a handful of cases documented in the literature (1, 2). In a typical anatomy, the LMCA arises from the left coronary sinus of Valsalva above the aortic valve. An origin from the LVOT implies that the coronary artery is exposed to subvalvular pressure, which can alter coronary perfusion dynamics and potentially compromise myocardial oxygen delivery.

To date, only a few reports have described the LMCA arising from the LVOT.

Such anomalies may be asymptomatic, as in our case, or may manifest with chest pain, dyspnea, syncope, arrhythmias, or even sudden cardiac death, particularly in young individuals or athletes. The first reported case was diagnosed at autopsy by Land et al. (3), involving a 15-year-old female athlete who died suddenly and was found to have an anomalous left coronary artery. The pathophysiology in these cases is complex and may involve myocardial ischemia due to reduced perfusion pressure, or coronary steal phenomenon due to abnormal communications. In the next report, Pirelli et al. (4) proposed an embryologic hypothesis involving an occlusive membrane, although this explanation remains unconvincing.

Oliveira et al. (5) reported a case of an asymptomatic middle-aged woman who was also incidentally diagnosed with an anomalous origin of the left coronary artery arising from the left ventricle. The patient had no symptoms, but the stress test was positive. Khan et al. (6) reported a case of a middle-aged woman who was admitted with acute chest pain and underwent coronary artery bypass grafting because of this abnormality. In the case reported by Wang et al. (7), the patient presented with marked biventricular dysfunction and also required surgical revascularization. Vlaar et al. (8) were the first to report both an anomalous origin and hypoplasia of the LMCA arising from the LVOT in young patients with stable chest pain, who underwent coronary artery bypass grafting.

In our case, the patient was discovered by chance, asymptomatic, and also elderly. LMCA-derived hypoplastic element is probably the factor that helps stabilize coronary perfusion. Significant dilation of the right coronary artery with collateral circulation from the RCA to the left coronary system explains the absence of ischemia despite the anomaly. In our case, asymptomatic condition in elderly patients was indeed a rare occurrence, emphasizing the role of LMCA ostium size in ensuring coronary perfusion from RCA collateral contribution. Table 1 provides an overview of documented case reports in the literature, arranged by year of publication.

**Table 1:** Published reports of an anomalous LMCA arising from the LVOT identified in the literature, listed by publication year.

Review of documented anomalous LMCA from the LVOT cases							
Author	Land	Pirelli	Oliveira	Khan	Vlaar	Wang	Huynh
Year	1994	2008	2009	2017	2019	2019	2025
Country	United States	United States	Brazil	United States	The Netherlands	New Zealand	Vietnam
Age (years)	15	51	54	51	35	44	67
Gender	Female	Male	Female	Female	Female	Male	Female
Symptoms sign	Sudden death	Chest pain and dyspnea	Asymptomatic	Acute chest pain	Stable chest pain	Dyspnea and leg swelling	Asymptomatic
Echocardiography		Preserved LVEF			Dilated LV with a mildly reduced LVEF	Severe reduced LVEF	Preserved LVEF
Stress test			Positive	Positive			Negative
Diagnosis		ICA, TEE, in operation	CCTA, ICA	CCTA, ICA	CCTA, ICA, CMR	CCTA, ICA	CCTA, ICA
Management		CABG	Not reported	CABG	CABG	CABG	No surgery
Outcome	Sudden death	Good		Good	Good	No improvement in cardiac function	Good

LVEF, left ventricular ejection fraction; CCTA, coronary computed tomography angiography; ICA, invasive coronary angiography; TEE, transesophageal echocardiogram; CMR, cardiac magnetic resonance; CABG, coronary artery bypass graft surgery.

Advanced imaging, particularly CCTA, plays a vital role in non-invasive delineation of the coronary anatomy and identification of such anomalies. The patient had no cardiovascular symptoms and signs, had a normal electrocardiogram, preserved ventricular function, and no ischemia, supporting a conservative approach. This treatment decision is appropriate for ACC/AHA guidelines for adult congenital heart disease, that surgical interventions may be considered in symptomatic individuals or those with ischemia documented by stress testing or perfusion imaging (9).

This case contributes to the growing awareness of coronary anomalies that may otherwise go undetected, emphasizing the importance of detailed cardiac imaging in preoperative assessments, especially in elderly patients undergoing non-cardiac surgery.

## 4 Conclusion

This report highlights a rare congenital anomaly of the left main coronary artery arising from the left ventricular outflow tract, discovered incidentally during preoperative evaluation in an asymptomatic patient. It underscores the value of comprehensive cardiac imaging and supports a conservative management strategy in the absence of symptoms and preserved cardiac function.

## Data Availability

The original contributions presented in the study are included in the article/Supplementary Material, further inquiries can be directed to the corresponding author.
